# A Connexin-Based Biomarker Model Applicable for Prognosis and Immune Landscape Assessment in Lung Adenocarcinoma

**DOI:** 10.1155/2022/9261339

**Published:** 2022-10-12

**Authors:** Junqing Qi, Jun Yin, Guowen Ding

**Affiliations:** ^1^Department of Cardiothoracic Surgery, Affiliated People's Hospital of Jiangsu University, Zhenjiang, China; ^2^Department of Cardiothoracic Surgery, Zhongshan Hospital, Fudan University, Shanghai, China; ^3^School of Medicine, Jiangsu University, Zhenjiang, China

## Abstract

**Purpose:**

Gap junction protein (Connexin) family is the basic unit of cellular connection, whose multiple members were recently demonstrated to be associated with tumor progression. However, the expression pattern and prognostic value of connexin in lung adenocarcinoma (LUAD) have not yet been elucidated.

**Methods:**

Consensus cluster algorithm was first applied to determine a novel molecular subtype in LUAD based on connexin genes. The differentially expressed genes (DEGs) between two clusters were obtained to include in Cox regression analyses for the model construction. To examine the predictive capacity of the signature, survival curves and ROC plots were conducted. We implemented GSEA method to uncover the function effects enriched in the risk model. Moreover, the tumor immune microenvironment in LUAD was depicted by CIBERSORT and ssGSEA methods.

**Results:**

The integrated LUAD cohort (TCGA-LUAD and GSE68465) were clustered into two subtypes (C1 = 217 and C2 = 296) based on 21 connexins and the clinical outcomes of LUAD cases in the two clusters showed remarkable discrepancy. Next, we collected 222 DEGs among two subclusters to build a prognostic model using stepwise Cox analyses. Our proposed model consisted of six genes that accurately forecast patient outcomes and differentiate patient risk. GSEA indicated that high-risk group was involved in tumor relevant pathways were activated in high-risk group, such as PI3K/AKT signaling, TGF-*β* pathway, and p53 pathway. Furthermore, LUAD cases with high-risk presented higher infiltration level of M2 macrophage and neutrophil, suggesting high-risk group were more likely to generate an immunosuppressive status.

**Conclusion:**

Our data identified a novel connexin-based subcluster in LUAD and further created a risk signature which plays a central part in prognosis assessment and clinical potency.

## 1. Introduction

Lung cancer is one of the most common malignancies worldwide, and the prevention of lung cancer is a worldwide public health issue. According to the latest statistics published by the International Agency for Research on Cancer (IARC), the global incidence and mortality rates of lung cancer in 2020 are among the highest in the world [1]. The risk of lung cancer will continue to intensify and become prominent within the future given the huge population base, aging, and high levels of tobacco consumption [2]. The incidence of lung adenocarcinoma (LUAD) is increasing every year and accounts for more than half of nonsmall cell lung cancer [3]. Front-line clinicians have been pushing for the promotion of new technologies for comprehensive treatment (such as radiotherapy, immunotherapy, and targeted therapy), which have greatly reduced intraoperative injuries and postoperative complications for LUAD patients, but the diagnosis and treatment of LUAD is still encountering critical challenges [4]. For example, most patients have obvious symptoms at the time of consultation. In addition, the high incidence of resistance to radiotherapy and immunotherapy has contributed to unfavorable clinical outcomes for patients. Lung cancer is a highly heterogeneous tumor, and its occurrence is the result of coregulation of multiple genes [5]. In-depth investigation of the molecular mechanism of LUAD will provide valuable guidance for early diagnosis and individualized treatment of LUAD.

Tumor-infiltrating immune cells (TIICs) are an integral part of the tumor microenvironment (TME), including tumor-associated macrophages (TAMs), lymphocytes, and natural killer (NK) cells [6]. These immunocytes play a central part in killing management of tumors (e.g. CD8+ T cells and NK cells) on the one hand and in fostering tumor development on the other. In view of its vital role in tumor progression, the TME has emerged as an essential therapeutic target [7]. Immunosuppression of CD8+ T cells within the TME can be relieved by the use of PD1 inhibitors. It has achieved remarkable effect on the treatment of melanoma, lymphoma, and other tumors, suggesting that immunotherapy holds favorable prospects [8]. However, most patients are still experiencing poor outcomes after immunotherapy. Therefore, the immune landscape of TME in LUAD needs to be further elucidated.

The gap junction (GJ) is a special membrane structure consisting of an arrangement of connecting channels between two adjacent cells. Gap junction proteins (Connexins) are the basic units of GJ formed mainly in the cell membrane and cytoplasm [9]. Connexin participates in the exchange of messages and substances between cells and serves as an important regulator of physiological processes such as cell metabolism, internal environment stability, proliferation, and differentiation. Posttranslational modifications of connexin are often precisely regulated by cellular signaling networks [10]. Studies have demonstrated that connexin is closely bound up with a variety of classical cellular signaling pathways including MAPK, TGF-*β*, and Wnt pathways [11, 12]. Previous findings indicated that tumor cells present defective gap junction communication and abnormal expression of gap junction protein (connexin, Cx) [13]. As the most widely expressed gene in the Cx family, Cx43 shows the closest relationship with tumors. Poyet et al. revealed that downregulation of Cx43 expression correlates with gastric cancer tissue type, tumor differentiation degree, and clinical stage [14]. In bladder cancer, overexpression of Cx43 boosts tumor cell survival and progression by reinforcing the activity of intercellular gap junctions [15]. Moreover, GJA1 was proved to be a target gene of miR-30b-5p which could contribute to pancreatic cancer angiogenesis [16]. Nevertheless, up to now, it remains very little research on the role of connexins in LUAD.

With the advent of histological technologies and big data analysis, researchers can obtain more detailed information from tumor cells and effectively identify complex molecular features of tumors from massive amounts of data, enabling a deeper understanding of tumor biological features and clinical phenotypes [17]. Advances in bioinformatic analysis technologies have permitted researchers to observe a panoramic view of the biological process of tumor progression directly through clinical samples, which has furthered our insights into the identification of novel multiple biomarker-based signatures for clinical prediction [18, 19]. Consequently, exploring important clinically relevant variables and validating their reliable correlation with patient prognosis is a pivotal factor in facilitating the evolution of precision tumor therapy.

In this academic research, the genetic characteristics of connexins in LUAD were detected according to the data from public databases. Furthermore, we determine a novel molecular subtype based on connexins and uncover the clinical potency of the connexin-related model in LUAD cases.

## 2. Methods

### 2.1. Data Collection

The gene expression profile and the corresponding clinical information were obtained from the GEO (https://www.ncbi.nlm.nih.gov/geo/) and TCGA (https://portal.gdc.cancer.gov/) databases, respectively. The LUAD cohort from the TCGA database containing the gene expression and the clinical information of 535 LUAD patients was utilized as the training set to establish the prognostic model, and the GSE68465 dataset containing RNA sequencing of 442 LUAD samples was selected as the validation set. The exclusion benchmarks were set as follows: (1) histologic diagnosis is not LUAD, (2) cases without completed data, and (3) overall survival time of less than 30 days. A total of 21 connexins were retrieved from previous research [20]. The gene information of all connexins is summarized in Supplementary Table 1.

### 2.2. Connexins Gene Cluster Analysis

A total of 21 connexins were subjected to determine the connexin-based molecular subtype using the R package “ConsensusClusterPlus” [21]. The difference between different subclusters was evaluated using the Kaplan–Meier survival analysis. The differentially expressed genes (DEGs) were screened by the “limma” package [22], before being processed for subsequent analysis.

### 2.3. Construction of Connexin-Related Signature

All the samples in training cohort were randomly divided into training and internal validation cohorts at a 1 : 1 ratio. Univariate Cox analysis was employed to discover prognostic genes in the training cohort. Next, the corresponding coefficients of these model genes were calculated to establish a prognostic model by multivariate analysis. The formula was established as follows: the risk score = ∑_*i*=1_^*n*^(coef × Exp_*i*_). The $\text{Ex}{\text{p}}_{\underset{¯}{i}}$ was the expression level of each gene and the coef was the risk coefficient of each gene. All the patients were divided into high- and low-risk group base on median risk value. To verify the predictive performance of the connexin-related gene signature, an external dataset, GSE68465, was enrolled into subsequent validation.

### 2.4. Immune Activity Analysis

The CIBERSORT algorithm (https://cibersort.stanford.edu/) was used to quantify the relative infiltration levels of 21 types of immune cells, as described before. The immune activity between the two risk subgroups, as demonstrated by normalized enrichment score (NES), were compared by the single sample gene set enrichment analysis (ssGSEA) [23].

### 2.5. Functional Enrichment Analysis

GSEA analysis was performed to reveal the potential molecular mechanisms of prognosis related genes and adjusted *p* < 0.05 was set as the cutoff value [24]. To obtain the signaling pathways for LUAD patients, the Kyoto Encyclopedia of Genes and Genomes (KEGG) was performed and visualized by the use of “clusterProfiler” and “ggplot2” R package, respectively.

### 2.6. Statistical Analysis

All statistical data in this research was analyzed by R version 4.0.5. In order to further assess the predictive capacity of the established signature, the Kaplan–Meier survival analysis was performed using the “survival” R package, and the time-dependent receiver operational feature curves (ROC) were drawn based on the “survival ROC” R packages. The area under the ROC (AUC) values for 1-, 3-, and 5-year survival rate were calculated. Univariate and multivariate Cox analyses were implemented to confirm the independence of the model.

## 3. Results

### 3.1. The Genetic Characteristics of Connexins

**Figure 1:**
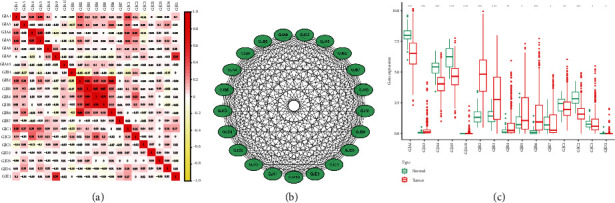
The genetic characteristics of Connexins in LUAD. (a) Correlation between expression levels of 21 connexins. (b) PPI network of 21 connexins. (c) Expression patterns of 21 connexins.

First, we detected the correlation between 21 connexins in TCGA-LUAD dataset. The results suggested that there was a significant coexpression relationship between GJA4 and GJA5, GJA9 and GJE1, and GJB2 and GJB6 (Figure 1(a)). To explore the interaction relationship of 21 connexins at protein level, a PPI network was set up by STRING tool (Figure 1(b)). As suggested by Figure 1(c), the GJA3, GJA10, GJB2, GJB3, GJB4, GJB5, and GJB6 were remarkably enriched in LUAD tissues, while GJA1, GJA4, GJA5, GJB7, GJC1, GJC2, GJC3, and GJD2 were greatly downregulated.

### 3.2. Determination of a Connexin-Based Molecular Subtype

**Figure 2:**
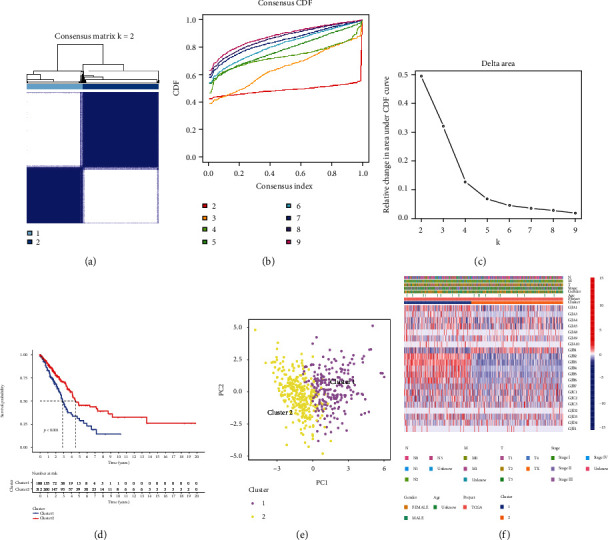
Connexin-based consensus clustering analysis. (a) Consensus cluster analysis. (b)-(c) Relative change of CDF curve. (d) The Kaplan–Meier survival analysis. (e) Principal component analysis of the two clusters. (f) Heatmap of connexin-related cluster.

With the 21 connexins included in consensus cluster analysis, we found that all LUAD cases were clustered into two subgroups (Figure 2(a)). The intergroup relationship between two subtypes was lowest when *k* = 2 (Figures 2(b) and 2(c). Survival curves illustrated that there were notable discrepancies in patient outcomes between two subgroups (Figure 2(d)). PCA analysis revealed two groups of significant cluster characteristics (Figure 2(e)). In Figure 2(f), there was a tight correlation between cluster and different clinical traits. Then, a total 222 DEGs were obtained between two clusters for next Cox analysis.

### 3.3. Construct of a Prognostic Signature

**Figure 3:**
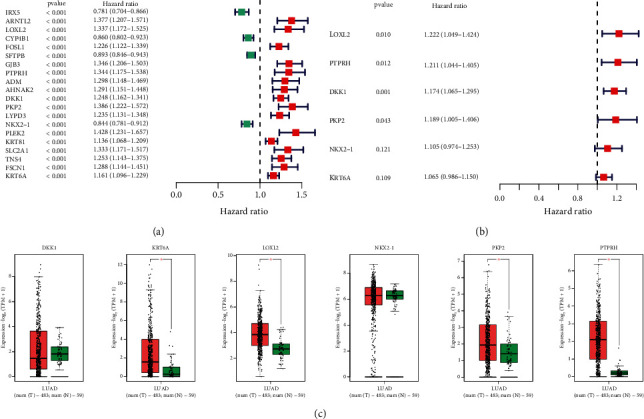
Development of a prognostic model. (a) Univariate Cox regression analysis. (b) Multivariate regression analysis for model construction. (c) Expression level of six model genes (LOXL2, PTPRH, DKK1, PKP2, NKX2-1, and KRT6A) from the GEPIA database.

In the training set, we first employed univariate Cox regression to discover 20 genes with prognostic values in LUAD (Figure 3(a)). Next, these 20 genes were enrolled into multivariate Cox regression and six model genes (LOXL2, PTPRH, DKK1, PKP2, NKX2-1, and KRT6A) were determined to create a prognostic model (Figure 3(b)). The risk score = (−0.2665 × LOXL2) + (0.1905 × PTPRH) + (0.1281 × DKK1) + (0.5798 × PKP2) + (0.4434 × NKX2 − 1) + (0.0103 × KRT6A). Then, we performed GEPIA database to explore the expression patterns of six model genes. As shown in Figure 3(c), LOXL2, PTPRH, PKP2, and KRT6A were greatly upregulated in LUAD tissues.

**Figure 4:**
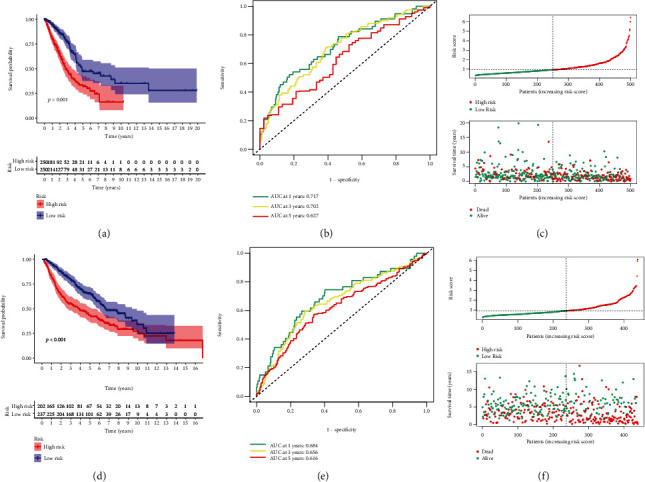
Predictive performance of the signature. (a) and (d) Survival analysis in the TCGA and the GEO datasets. (b) and (e) ROC curves of the signature. (c) and (f) The risk distribution plots in two independent cohorts.

Survival analysis indicated that patients with high-risk displayed a dismal clinical outcome (Figure 4(a)). The AUC (area under the curve) values of 1-, 3-, and 5-year survival rate generated by the model were 0.717, 0.702, and 0.627, respectively (Figure 4(b)). The risk plot of six genes signature is shown in Figure 4(c). Moreover, the same methods were conducted in GSE68465 cohort to confirm the performance of the model, and the similar results were observed (Figures 4(d)–4(f)).

### 3.4. Independent Prognostic Analysis and Subgroup Analysis

**Figure 5:**
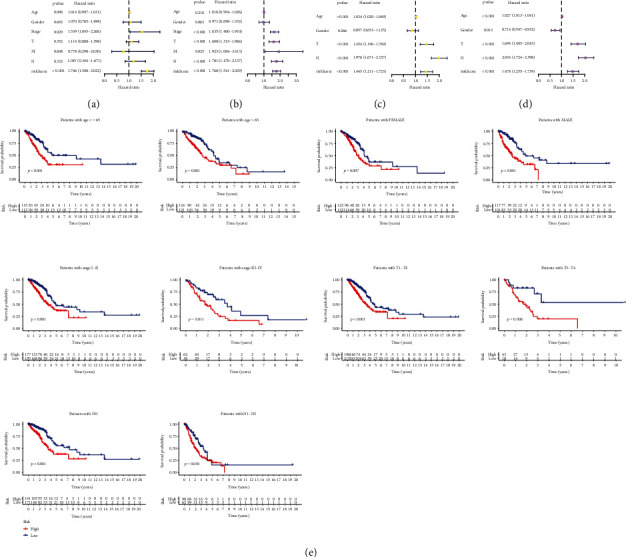
Independent prognosis analysis. (a) and (c) Univariate regression analysis the TCGA and the GEO datasets. (b) and (d) Multivariate regression analysis in two cohorts. (e) Subgroup analysis of the risk model based on age, gender, stage, T stage, and N stage.

To examine the independence of the risk model, univariate and multivariate methods were applied. In the TCGA cohort, univariate analysis showed that stage and the risk score were hazard factors for evaluating patient outcome (Figure 5(a)). Multivariate Cox analysis showed that risk score (*p* < 0.001) was independent factor for assessing prognosis of LUAD (Figure 5(b)). Meanwhile, the independence of our established signature was validated in the GSE68465 cohort (Figure 5(c) and 5(d)). Next, we further detected whether the risk model is a prognostic factor for the survival assessment in different subgroups with various clinical traits. In Figure 5(e), the survival rates of the high-risk patients based on age, gender, stage, T stage, and N stage were lower than those of the low-risk patients.

### 3.5. GSEA of the Risk Model

**Figure 6:**
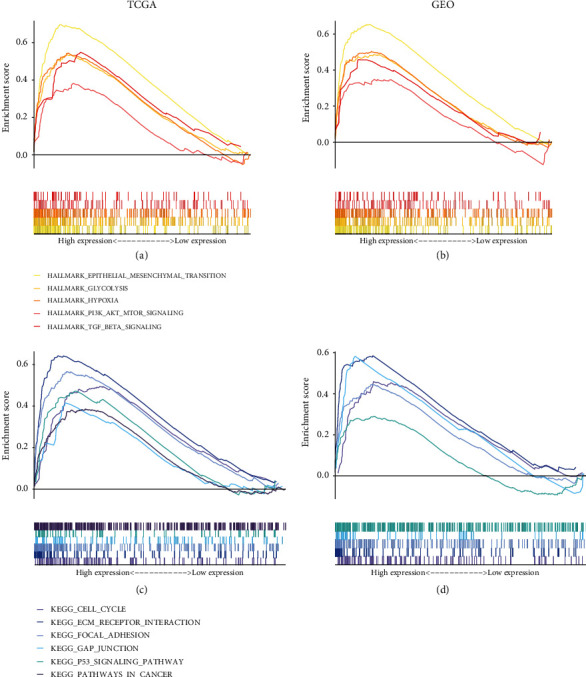
Gene set enrichment analysis. (a) and (b) Hallmark analysis of the two risk groups. (c) and (d) KEGG analysis of the two risk groups.

GSEA showed that top five Hallmarks were greatly enriched in high-risk group, including epithelial-mesenchymal transition, glycolysis, hypoxia, PI3K/AKT/MTOR signaling, and TGF-*β* signaling (Figures 6(a) and 6(b)). KEGG analysis revealed that high-risk group was positively correlated with pathway in cancer, cell cycle, and p53 pathway (Figures 6(c) and 6(d)).

### 3.6. The Immune Landscape of LUAD

**Figure 7:**
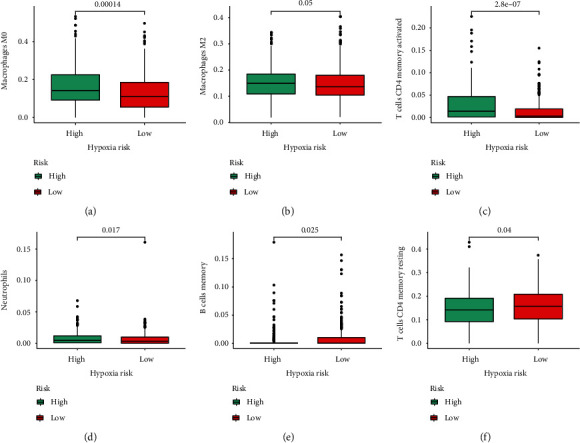
Immune infiltration analysis. (a) Macrophages M0. (b) Macrophages M2. (c) Activated CD4 memory T cells. (d) Neutrophils. (e) Memory B cells. (f) Resting CD4 memory T cells.

**Figure 8:**
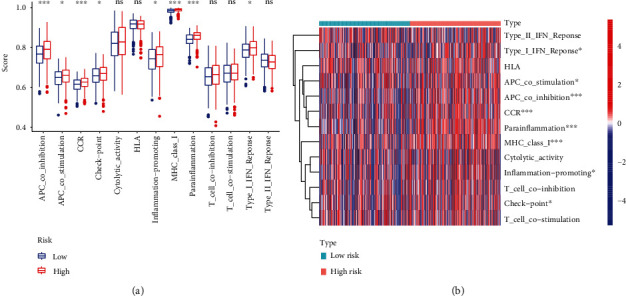
Immune function analysis. (a) The boxplot demonstrated the differences in immune function between two groups. (b) Heatmap of immune function analysis (^∗^*p* < 0.05; ^∗∗^*p* < 0.01; ^∗∗∗^*p* < 0.001).

In order to characterize the immune microenvironment of LUAD cases, we first calculate the proportion of 21 different immunocytes by CIBERSORT algorithms. The results revealed that macrophages M0, macrophages M2, activated CD4 memory T cells, and neutrophils were enriched in high-risk cohort, whereas memory B cells and resting CD4 memory T cells were upregulated in low-risk cohort (Figure 7). Furthermore, we compared the difference in immune activity between the two groups by ssGSEA. As revealed by Figure 8, APC-related function, immune checkpoints, inflammation−promoting, and IFN type II were activated greatly in high-risk groups.

## 4. Discussion

LUAD is one of the most frequently diagnosed malignancies globally and is currently the leading cause of cancer death [25, 26]. Jemal et al. once reported that nearly 70% of LUAD patients were discovered at terminal stages at the first time of diagnosis, with 60% of them already developed distant metastasis by then [27]. Although great efforts have been made in the exploration of gene mutation targeted therapy, the five-year survival rate of LUAD patients remain dismal, which is mainly due to the lack of specific and reliable biomarkers [28]. Taken together，it is urgently needed to explore effective and less invasive surrogate molecular biomarkers that can help determine the clinical outcome of LUAD patients and further develop more promising therapeutic targets for cancer treatment.

Connexin hemichannels have long been recognized as structural precursors to form gap junctions [29]. Thus, connexins play important role in the maintaining tissue homeostasis, and their mutation is implied to induce the onset of multiple disorders. Following that, accumulating evidence has unveiled the involvement of connexins in carcinogenesis, including prostate cancer, renal cancer, and glioma cancer [30–33]. Intriguingly, connexins are reported to have distinct expression patterns at different stages of tumor progression. More specifically, connexins showed declined expression in the primary stage, while can be an overexpression when tumor cells developed a more invasive phenotype [34]. Until now, the understanding of connexin channels in LUAD is rather restrained [35, 36]. In the current study, we divided LUAD into two distinct subtypes based on expression profiles of 21 types of connexins. In principle, LUAD patients in cluster 1 showed much poorer outcome compared to their counterparts in cluster 2. Subsequently, we identified 222 DEGs between the two populations for the establishment of prognostic model.

Advances in “Next-generation” sequencing technology have laid the foundation for the development of gene signatures in which clinical outcome of patients can be assessed on the basis of their transcriptomic data as well as the pathological grading. A total of six-gene based were verified to play a critical role in predicting clinical outcome patients with LUAD. LOXL2, which is strongly induced by hypoxia condition, has been identified to exert its protumor effects by promoting tumor progression in various cancers, including breast cancer, colorectal cancer, cervical cancer, and LUAD [37, 38]. In LUAD, LOXL2 was demonstrated to contribute to cell surface matrix remodeling and subsequently bring dissemination of tumor cell aggregates [39]. Protein tyrosine phosphatases (PTP) family is well known for its role in regulating tumor cell proliferation, migration, and invasion in pathology of cancers. Chen et al. once validated the prognostic value of PTPRH in LUAD tissues. The transcription as well as the protein level of PTPRH was found to be noticeably upregulated in LUAD tissues, as demonstrated by qRT-PCR and immunohistochemistry, respectively [40]. The role of DKK1 in cancer development remains unelucidated. Although DKK1 has been reported to act as a tumor suppressor in various malignant tumors, opposing results regarding DKK1 expression and its role in cancer have been achieved recently [41, 42]. For instance, Zeybek et al. reported that the expression levels of the DKK1 in early-stage LUAD tissue were significantly downregulated compared to their counterparts in normal tissues and were closely related to the tumor progression [43]. Aberrantly expressed PKP2 has been found in a number of tumors, including bladder, osteosarcoma, and ovarian cancers [44, 45]. GSEA analysis revealed that PKP2 expression is positively associated with EGFR signaling in LUAD. It is worth noting that studies regarding the precise functions of these genes in regulating the development of LUAD remain rare until now, further research should focus on elucidating their biological functions on the basis of our work.

Molecular mechanisms participating in the regulation of LUAD were validated using the GSEA analysis. Top five Hallmarks including “EMT”, “hypoxia”, “glycolysis” “PI3K/AKT”, and “TGF-*β*” were observed to be associated with the prognosis of LUAD patients in our gene signature. Activation of EMT, characterized by the loss of cell polarity and the breakdown of basement membrane, can bring mesenchymal characteristics to epithelial cells and finally promote tumor metastasis [46, 47]. EMT can also interact with “hypoxia” and “glycolysis” signaling to induce metabolic reprogramming in cancer cells [48]. The tumor progression renders the nutrients limited supply. As a result, tumors attempt to adapt to the hypoxia TME by switching to glycolysis from mitochondrial oxidative phosphorylation for their energy production, which is now known as the Warburg effect [49]. The involvement of genetic alterations of PI3K/AKT signaling in promoting the onset and development of LUAD has been largely reported [50]. In line with previous studies, the PI3K/AKT pathway was found to be aberrantly activated in high-risk LUAD patients. Altogether, these hallmarks represent attractive therapeutic targets for the detection of novel anticancer therapies.

Additionally, we determined the distinct immunocyte infiltration status in high- and low- risk LUAD patients. M0 macrophages, M2 macrophages, activated CD4 memory T cells, and neutrophils were enriched in high-risk patients, whereas memory B cells and resting CD4 memory T cells were relatively abundant in low-risk patients. Our results revealed that there may be some existing interactions between the expression pattern of connexins and infiltration situation in LUAD patients, which sheds lights on the detection of novel tumor immunotherapy.

Numerous reports have demonstrated a tight relationship between inflammation and cancer. The inflammatory component of tumor development involves a various population of leukocytes. These immune cells could be served as a crucial inflammatory contributor to cancer progression by releasing cytokines, chemokines, and cytotoxic mediators. Cancer-associated inflammation has impact on malignancies in many ways, including cell growth, cancer metastasis, and therapeutic resistance [51]. Although short-term IFN-*γ* stimulation can enhance the expression of MHC class I and antigen presentation in tumor cells, prolonged IFN-*γ* exposure may lead to immune escape. On the one hand, tumor cells can reduce the IFN-*γ*-dependent immunosurveillance by affecting the expression and activity of IFN-*γ*, leading to the occurrence of immune escape. Also, IFN-*γ* can activate crucial immune escape genes such as PD-L1 and CTLA-4 [52]. In our data, we found that promoting inflammation and IFN response were activated in high-risk group, suggesting patients are prone to be immunosuppressive status. In addition, LUAD cases with high-risk may benefit from Immuno-Checkpoint Inhibitor (ICI) since these patients presented higher expression of immune checkpoints.

However, there are some limitations in our analysis. The data for building the model were mainly from public databases. Although the model has been confirmed in two independent datasets, its reliability still needs further validation in more real-world cohorts. The expression patterns of six model genes should be detected based on clinical LUAD specimens. Moreover, various experiments need to be conducted to explore the underlying molecular functions and mechanisms of connexin-related biomarkers. In the present study, we observed that the risk model displayed robust predictive power for assessing patient outcomes and could be stably applied to patients with LUAD. Furthermore, our constructed model could be served as a predictor for mirroring immune status of LUAD cases and provide valuable reference for therapeutic strategies.

In conclusion, we established an effective prognostic model consist of six genes on the basis of connexins molecular subtypes. Molecular signaling, immune phenotypes, and immune activities in two risk cohorts were further assessed. Taken together, our gene signature can help provide potential therapeutic targets for the different subclusters of LUAD patients and may aid in helping them choose personalized immunotherapy.

## Data Availability

The public datasets to support the results of this research can be collected from TCGA (https://portal.gdc.cancer.gov/) and GEO (https://www.ncbi.nlm.nih.gov/geo/).

## Supplementary Materials

Supplementary Materials Supplementary Table 1: the list of 21 connexin genes.
