# Supplementary material for: A Connexin-Based Biomarker Model Applicable for Prognosis and Immune Landscape Assessment in Lung Adenocarcinoma
Source: J Oncol. 2022 Oct 12;2022:9261339. doi: 10.1155/2022/9261339 (PMC9581606; doi:10.1155/2022/9261339)
Supplement: Supplementary Materials — Supplementary Table 1: the list of 21 connexin genes. [file 9261339.f1.docx]

**Supplementary Table 1** The list of 21 connexin genes.

| Gene Symbol | Description | Category | Gifts | GC Id | Relevance score |
| --- | --- | --- | --- | --- | --- |
| GJA1 | Gap Junction Protein Alpha 1 | Protein Coding | 48 | GC06P121436 | 51.60049 |
| GJA10 | Gap Junction Protein Alpha 10 | Protein Coding | 37 | GC06P089894 | 12.02122 |
| GJA3 | Gap Junction Protein Alpha 3 | Protein Coding | 41 | GC13M020474 | 21.91289 |
| GJA4 | Gap Junction Protein Alpha 4 | Protein Coding | 42 | GC01P034792 | 23.64044 |
| GJA5 | Gap Junction Protein Alpha 5 | Protein Coding | 44 | GC01M147756 | 29.25836 |
| GJA8 | Gap Junction Protein Alpha 8 | Protein Coding | 44 | GC01P147902 | 21.77026 |
| GJA9 | Gap Junction Protein Alpha 9 | Protein Coding | 35 | GC01M038874 | 12.53584 |
| GJB1 | Gap Junction Protein Beta 1 | Protein Coding | 45 | GC0XP071212 | 37.30913 |
| GJB2 | Gap Junction Protein Beta 2 | Protein Coding | 45 | GC13M020187 | 58.77079 |
| GJB3 | Gap Junction Protein Beta 3 | Protein Coding | 43 | GC01P034781 | 33.1715 |
| GJB4 | Gap Junction Protein Beta 4 | Protein Coding | 38 | GC01P034759 | 21.02325 |
| GJB5 | Gap Junction Protein Beta 5 | Protein Coding | 39 | GC01P034755 | 16.0032 |
| GJB6 | Gap Junction Protein Beta 6 | Protein Coding | 42 | GC13M020221 | 38.74098 |
| GJB7 | Gap Junction Protein Beta 7 | Protein Coding | 31 | GC06M087282 | 11.84414 |
| GJC1 | Gap Junction Protein Gamma 1 | Protein Coding | 40 | GC17M044949 | 20.37275 |
| GJC2 | Gap Junction Protein Gamma 2 | Protein Coding | 40 | GC01P229296 | 14.14757 |
| GJC3 | Gap Junction Protein Gamma 3 | Protein Coding | 37 | GC07M099923 | 13.08359 |
| GJD2 | Gap Junction Protein Delta 2 | Protein Coding | 40 | GC15M034751 | 15.30465 |
| GJD3 | Gap Junction Protein Delta 3 | Protein Coding | 32 | GC17M040360 | 11.0729 |
| GJD4 | Gap Junction Protein Delta 4 | Protein Coding | 35 | GC10P035611 | 10.26779 |
| GJE1 | Gap Junction Protein Epsilon 1 | Protein Coding | 22 | GC06P142133 | 6.702111 |
